# Automated three-dimensional image registration for longitudinal photoacoustic imaging

**DOI:** 10.1117/1.JBO.29.S1.S11515

**Published:** 2024-01-13

**Authors:** Bruno De Santi, Lucia Kim, Rianne F. G. Bulthuis, Felix Lucka, Srirang Manohar

**Affiliations:** a University of Twente, TechMed Centre, Multi-Modality Medical Imaging Group, Enschede, The Netherlands; b Medisch Spectrum Hospital, Department of Radiology, Enschede, The Netherlands; c Centrum Wiskunde en Informatica (CWI), Amsterdam, The Netherlands

**Keywords:** photoacoustic imaging, breast imaging, longitudinal imaging, image registration, coordinate based neural network

## Abstract

**Significance:**

Photoacoustic tomography (PAT) has great potential in monitoring disease progression and treatment response in breast cancer. However, due to variations in breast repositioning, there is a chance of geometric misalignment between images. Further, poor repositioning can affect light fluence distribution and imaging field-of-view, making images different from one another. The net effect is that it becomes challenging to distinguish between image changes due to repositioning effects and those due to true biological variations.

**Aim:**

The aim is to develop a three-dimensional image registration framework for geometrically aligning repeated PAT volumetric images, which are potentially affected by repositioning effects such as misalignment, changed radiant exposure conditions, and different fields-of-view.

**Approach:**

The proposed framework involves the use of a coordinate-based neural network to represent the displacement field between pairs of PAT volumetric images. A loss function based on normalized cross correlation and Frangi vesselness feature extraction at multiple scales was implemented. We refer to our image registration framework as MUVINN-reg, which stands for multiscale vesselness-based image registration using neural networks. The approach was tested on a longitudinal dataset of healthy volunteer breast PAT images acquired with the hybrid photoacoustic-ultrasound Photoacoustic Mammoscope 3 imaging system. The registration performance was also tested under unfavorable repositioning conditions such as intentional mispositioning, and variation in breast-supporting cup size between measurements.

**Results:**

A total of 13 pairs of repeated PAT scans were included in this study. MUVINN-reg showed excellent performance in co-registering each pair of images. The proposed framework was shown to be robust to image intensity shifts and field-of-view changes. Furthermore, MUVINN-reg could align vessels at imaging depths greater than 4 cm.

**Conclusions:**

The proposed framework will enable the use of PAT for quantitative and reproducible monitoring of disease progression and treatment response.

## 1 Introduction

In photoacoustic (PA) imaging, short-pulsed near-infrared light is applied to tissue. Various tissue chromophores absorb the light to trigger thermoelastic expansion and pressure wave generation. Detection of these waves, which have frequencies in the ultrasound (US) regime, allows for image reconstruction of the absorbed optical energy.^1^ A strongly absorbing chromophore is hemoglobin, which enables PA imaging to accurately depict blood vessels. Further, due to the specific absorption spectra of hemoglobin in its two states, oxygenated and deoxygenated, the method can potentially report on blood oxygen saturation in tissue. It is known that vascularization associated with cancer is more abundant and structurally different than in healthy tissues, and oxygen saturation is an important physiological biomarker in tumor hypoxia. This makes PA imaging highly attractive for oncology.^2^ Furthermore, the method is noninvasive, contrast agent-free, relatively cheap, and with image acquisition times lower than conventional magnetic resonance imaging (MRI) protocols.

In the context of breast cancer, PA tomography (PAT) has shown encouraging results with multiple proposed devices exploiting different light delivery systems and US detection geometries.^3^ Promising PAT applications for breast cancer span tumor detection,^4^ diagnosis,^5^^,^^6^ and neoadjuvant chemotherapy (NAC) monitoring.^6^^,^^7^ The latter is a systemic therapy administered before surgery, with the goals of (1) reducing tumor size enabling breast-conserving surgery; (2) treating possible future metastatic disease, even if undetectable in preoperative staging; and (3) tailoring future chemotherapeutic decisions.

It is important to monitor response to NAC, to spare nonresponding patients the toxicity (and expenses) of ineffective treatments, and to prevent further tumor progression due to postponed surgery. Repetitive imaging at optimal time-points during NAC is conducted using MRI techniques, radionuclide (positron emission tomography) imaging, and hybrid imaging.^8^ This longitudinal imaging enables tumor assessment by evaluating the changes in size, shape, and characteristics of the tumor, and helps to gauge the effectiveness of NAC. Compared to the standard of care imaging modalities, PAT has advantages for repeat measurements and could play an important role in NAC response monitoring by providing a safe and noninvasive method to visualize biological and functional response in the tumor microenvironment.

One of the requirements in longitudinal imaging is that data between imaging sessions are comparable, from the point of view of patient positioning. In particular in PAT, repositioning of the breast inside the recording aperture can cause changes in the field-of-view as well as geometric misalignment of blood vessels between PAT scans, which challenges the measurement of qualitative and quantitative vascular changes.^9^ An available solution to this problem is the use of breast-supporting cups, which partially enable reproducible repositioning, but their use is inherently prone to human error and dependent on the operator’s experience.^9^ Further, such solutions can be complex to implement due to large variability in breast size, morphology, and composition.

Approaches based on image co-registration can constitute an effective and easy-to-implement solution to the problem of geometric misalignment between repeated scans. In this case, PAT scans acquired before and at different time points during the therapeutic regimen can be aligned in the same reference coordinate system such that the same anatomical structures become geometrically matched. This would allow the radiologist to conveniently focus on observing locoregional evolution in time or also, to develop and train computer-aided systems to extract changes in quantitative imaging biomarkers to monitor or predict the tumor response.

Several image registration algorithms have been proposed for PA imaging but most of them aimed at registering PA images with conventional imaging modalities such as MRI.^6^^,^^10^^–^^13^ To the best of our knowledge, only two research studies deal with the problem of unimodal registration of serial PA images.^14^^,^^15^ A parametric approach using an intensity-based scheme and gradient descent optimizer was proposed to co-register PAT images of hands.^14^ However, one major limitation of parametric registration algorithms is the requirement to choose the anticipated deformation field parametrization (examples are rigid, affine, or B-splines). This can be problematic in the case of complex and unknown deformations, i.e., the breast repositioning within the recording aperture. In a second study,^15^ a registration method based on PAT image decomposition (into foreground, noisy background, and corrupted foreground) and coarse-to-fine alignment was proposed. This was intended to correct body motion in *in vivo* PAT images by co-registering multiple shot-volumes before averaging.^15^ Integrating image decomposition with a multiscale approach was a key to cope with the sparse nature of PAT images, presence of noise, and variety in sizes of blood vessels. However, the algorithm is only able to correct for global affine transformations reducing its use to a limited number of applications. Several deep learning approaches have been proposed for medical image registration.^16^ However, most of these require a pretraining phase, and their generalization ability strongly depends on the amount of available training data. This makes their application inconvenient in PAT image registration owing to the general paucity of standardized datasets. Therefore, we believe that there is a strong need in this research field for an image registration framework able to align *in vivo* three-dimensional (3D) PAT images affected by complex and nonlinear deformations.

In this work, we propose a registration framework based on the use of coordinate-based neural networks and multiscale Frangi vesselness filtering to co-register longitudinal 3D PAT scans. The algorithm does not require any training data, *a priori* parametric deformation model, or manual landmark annotation. Following the recent study by Wolterink et al.,^17^ a multilayer perceptron (MLP) network is used to transform coordinates of the fixed image domain to the moving image domain, and hence, implicitly represent deformation fields. Thanks to the use of sinusoidal activation functions, the network is able to represent local and nonlinear deformation fields. We show the effectiveness of the framework in aligning repeated PAT scans acquired with the recently developed hybrid PA-US system [Photoacoustic Mammoscope 3 (PAM3)]^18^ under various conditions such as different illumination wavelengths and different breast supporting cups. Comparison with conventional image registration approaches shows the superiority of the proposed framework in serial PAT image alignment. We refer to the proposed framework as MUVINN-reg, which stands for multiscale vesselness-based image registration using neural networks. The proposed framework is implemented in Python and fully available together with data on GitHub (available at https://github.com/brunodesanti/muvinn-reg).

## 2 Background

### 2.1 Image Registration Algorithm

#### 2.1.1 Image registration as a minimization problem

The image registration problem can be defined as finding the optimal transformation Φ^ such that

(M∘Φ^)(x)=F(x), (1)

where $M:\Omega_{M}\subset R^{n}\to R$ and $F:\Omega_{F}\subset R^{n}\to R$ are the moving and fixed image, respectively, and $\Omega_{M}$ and $\Omega_{F}$ are the moving and fixed image domains, respectively. The optimal transformation can be found by solving the following minimization problem:

Φ^=arg minΦ Ldata(M∘Φ,F)+αLreg(Φ), (2)

where $L_{\text{data}}$ is a similarity measure between two images, $L_{\mathrm{reg}}$ is a regularization term to encourage regular deformation field, and α is a non-negative weighting parameter.

#### 2.1.2 Coordinate-based networks and their use for image registration

According to the universal approximation theorem, any continuous function can be approximated by a multilayer neural network with nonlinear activation functions.^19^ This allows a MLP network to represent a signal (e.g., image) by mapping its coordinate domain (x and y pixel coordinates) to the signal intensity (pixel intensity values). These networks can be referred to as coordinate-based neural networks and are the basis of a fast growing category of deep learning techniques called implicit neural representations (INRs). Excellent results have been demonstrated using INRs in many applications such as scene rendering,^20^ image reconstruction,^21^ and recently image registration.^17^^,^^22^

In image registration, an MLP network can be optimized to represent the displacement field u(x) such that coordinate x in the fixed image geometrically corresponds to coordinate ϕ(x)=u(x)+x in the moving image.^17^ A loss function based on a data term and a regularization term is used to maximize similarity between the images and encourage smooth displacement fields [see Eq. (2)]. The network is trained by using batches of coordinates from the fixed image domain as input. Using coordinate-based networks for image registration has several advantages compared to traditional or convolutional-based approaches. First, they allow to represent the deformation field continuously over the image domain with a low and fixed number of parameters (network weights). Second, they do not require any *a priori* information regarding the transformation model. Third, they allow derivatives to be computed analytically making the regularization task free of finite differences computations. Finally, these methods are highly flexible and extendable to different registration tasks, by changing the similarity metric, regularization term, or activation function.

### 2.2 PAM3 Imaging System

In this study, the hybrid PA-US PAM3 imaging system was used.^18^ The system is embedded in a custom-designed bed, which includes the laser system and the hemispherical US recording aperture. The subject lies prone on the bed with her breast positioned within the imaging bowl (26 cm diameter). The bowl accommodates the water-coupled US aperture, which comprises 512 single-element US transducers flush with the inside of the bowl. For PA excitation, the bowl has 40 optical fiber bundle terminations distributed on the inner surface of the bowl. Acquisition parameters such as number of bowl rotations, number of wavelengths, number of averages, and number of US shots can be programmed for each individual acquisition. Eight breast-supporting cups with different sizes are available and used to stabilize the breast during the measurements. The operator chooses the appropriate cup size before the acquisition.^18^ An iterative full-wave model-based image reconstruction method is to reconstruct PA images, which can make use of different speed of sound (SOS) models.^23^ Number of iterations was equal to 10 and voxel size equal to 0.4 mm.^18^ In the present work, we used a 2-SOS model with the known SOS for the coupling water and a single SOS for the whole breast, which is manually chosen to maximize the sharpness of the reconstructed blood vessels.

## 3 Methods

### 3.1 MUVINN Image Registration Framework

**Fig. 1:**
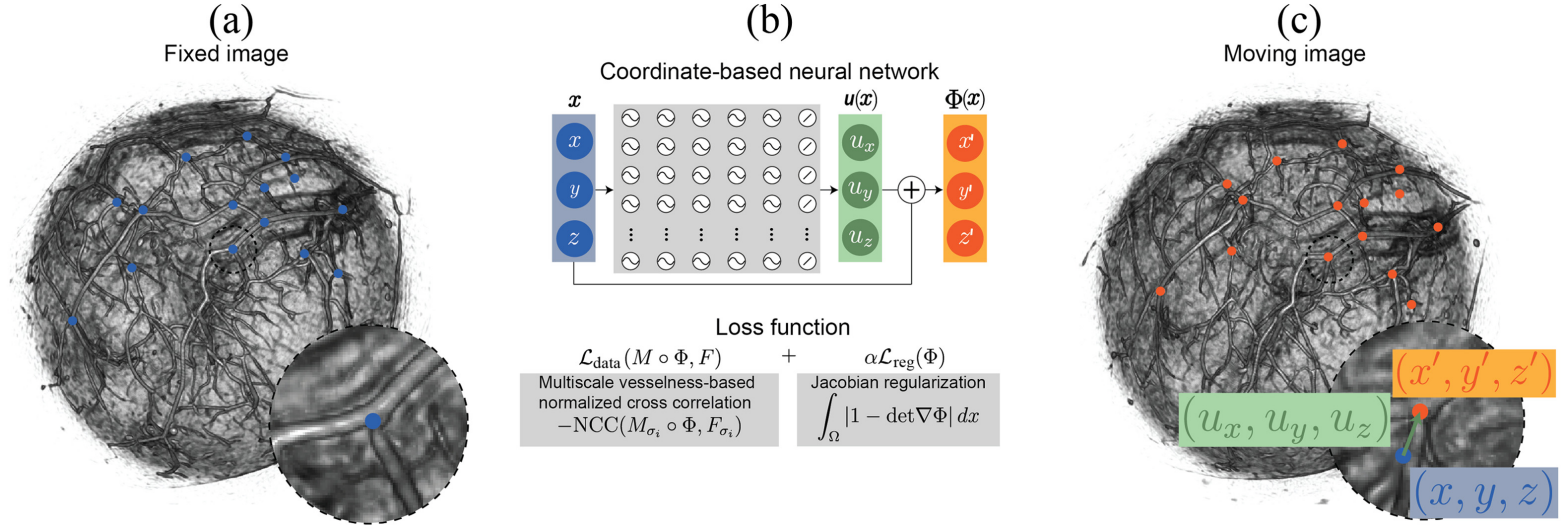
Overview of MUVINN image registration framework. (a) Coordinate sampling from the fixed image domain. (b) MUVINN uses a coordinate-based MLP with sinusoidal activation functions to represent the displacement field, u(x) such that transformed coordinates Φ(x)=x+u(x) on the moving image anatomically correspond to coordinates x. in the fixed image. NCC between multiscale Frangi vesselness filtered images are used as data similarity term. Jacobian regularization is used to find smooth and regular deformation fields. (c) Coordinate transformation into the moving image domain.

The proposed MUVINN framework aims to co-register serial PAT scans of the breast. A coordinate-based MLP is optimized to find the displacement field between a first or reference PAT scan called the fixed image, and a second PAT scan called the moving image, acquired after breast repositioning inside the recording aperture. The framework is based on Wolterink et al.^17^ who proposed the use of coordinate-based neural networks for co-registration of inspiration and expiration 3D computed tomography lung images. The algorithm was modified to adapt its use on PAT images of the breast, mainly by implementing a coarse-to-fine strategy with the use of multiscale Frangi vesselness filtering within the network optimization. An overview of the proposed framework is shown in Fig. 1. The entire framework was implemented in CUDA-enabled PyTorch.

#### 3.1.1 Coarse-to-fine similarity term

In the context of PAT image registration, vascular structures are a valuable source of common information, since PAs are sensitive to blood. For this reason, the similarity metric was computed on Frangi vesselness filtered versions of the original images. A coarse-to-fine strategy was adopted to favor alignment of vessels with different sizes; network optimization starts with a higher value of the standard deviation σ of the gaussian kernel to enhance only the main vascular structure, then, σ is progressively decreased in a step-wise fashion during training iterations to enhance smaller vessels. Using a higher σ provides the network with low noise and less sparse (in spatial domain) data in the first iterations, which has the positive effects of improving robustness to noise and improving gradients for the network optimization (see section 1 in the Supplementary Material). For each experiment, five values of sigma were used during optimization, σ={12,9,5,3,2}, 4000 training iterations each. Intensities of the Frangi filtered images were adaptively modulated to improve image contrast.^24^^,^^25^ Normalized cross correlation (NCC), which is more robust compared to intensity difference-based metrics such as L1 or mean square error, was used as the similarity metric to intensity shifts that might be caused by changes in light fluence due to breast repositioning.

#### 3.1.2 Regularization and activation function

Image registration is an ill-posed problem, hence, regularization is fundamental to avoid instabilities and converge to smooth deformation fields.^26^ Jacobian regularization was used to penalize excessive expansion and shrinkage of vascular features. It is reasonable to assume that breast repositioning will not heavily affect the size of biological structures in the tissue. Furthermore, any expansion and shrinkage is of interest when capturing biological changes over time due to therapy or disease progression, thus, not to be corrected. The weighting parameter α was set to 0.95 for all experiments.

Activation functions of the MLP have a direct effect on the ability of the network to accurately represent the signal (in this case, the displacement field). It has been shown that sinusoidal activation function has a superior ability to represent high frequency content than rectified linear unit.^27^ To correct for small and local misalignments of vessels, sinusoidal activation functions were employed in this study with an angular frequency ω equal to 30.

#### 3.1.3 Training and implementation details

As usually done for INRs,^27^ image coordinates were normalized in the range ${\left[-1,\;1\right]}^{3}$. A coarse segmentation mask of vessels was obtained on the fixed image by Frangi vesselness filtering σ=[2,3, and 4] and adaptive thresholding (threshold map exponentially decaying as a function of depth). In each training epoch, a batch of 200 coordinates was randomly sampled from the set of coordinates belonging to the mask. A 5×5×5 cubical patch was defined leading to a total number of $200\cdot 5^{3}=\;25,000$ coordinates for each training epoch. The side length of the patch was changed according to the scale of the Frangi filtering in the iteration, according to $2.5\cdot \sigma_{c}/100$ (in normalized units). This allowed to compute NCC in local patches with a size depending on the enhanced vessels around the sampled points during the optimization process. After transforming the coordinates through the network, intensity values were sampled from the fixed and moving images by linear interpolation and the loss was computed. Regarding model hyperparameters, an MLP was used with six hidden layers, each of which contained 300 units with sinusoidal activation function (except for the last one which uses linear activation functions to ensure linear mapping). Adam optimizer with learning rate of 5e−5 and 20,000 epochs was used. Training and tests were done on a Windows 11 machine with an Intel Core i9-11900K @ 3.5 GHz, 128 GB RAM and NVIDIA RTX3090 24 GB.

### 3.2 Experimental Protocol

The study was approved by the Medical Ethics Review Board and acknowledged by the Dutch Central Committee for Research on human subjects. A 59-year-old woman volunteered in the study. She was classified with Fitzpatrick skin type 2 and wore brassiere size 80E. The volunteer was informed about the study, and informed consent was obtained.

#### 3.2.1 Imaging session

A total of 15 scans were acquired in the imaging session. The imaging session was divided into two phases. In phase A, seven repeated PAM3 scans (S1 to S7) of the left breast were performed by using the breast supporting cup with the optimal size equal to 7 out of 8 (largest size). The optimal cup size was determined through test-fitting various cup sizes on the volunteer by the operator (author R.F.G.B.) prior to the start of the imaging session. Between each pair of repeated scans, the volunteer was asked to stand up and then lie down on the imager to reposition the breast in the cup. Scans S1 to S3 were acquired with dual wavelength (720 and 870 nm) while the remaining four (S4 to S7) were acquired with a single wavelength (720 nm). For scans S6 and S7, the breast was deliberately mispositioned in the cup to test the algorithm performance when a more severe deformation occurs. In phase B, the right breast was scanned eight times (S8 to S15) with a single wavelength (720 nm) using different breast-supporting cups (S8 and S9: cup size 7, S10 and S11: cup size 5, S12 and S13: cup size 4, and S14 and S15: cup size 8).

#### 3.2.2 MUVINN-reg registration experiments

The performances of the proposed image registration framework were evaluated under different serial imaging scenarios: experiment 1–standard breast repositioning; experiment 2–breast repositioning and different illumination wavelength; and experiment 3–breast repositioning and different breast-supporting cups. Table 1 shows an overview of the registration experiments conducted.

**Table 1:** Overview of the MUVINN-reg registration experiments. For simplicity, the same fixed scan was used within the same experiment.

		Scan	Wavelength (nm)	Cup size
Experiment 1: breast repositioning	Fixed image	S1	720	7
	Moving image(s)	S2 to S5 (normal repositioning)	720	7
		S6 and S7 (deliberate mispositioning)		
Experiment 2: breast repositioning with different illumination wavelength	Fixed image	S1	720	7
	Moving image(s)	S2 and S3	870	7
Experiment 3: breast repositioning with different breast-supporting cup	Fixed image	S8	720	7
	Moving image(s)	S10^a^ and S11	720	5
		S12 and S13	720	4
		S14 and S15	720	8

a S10 went missing due to technical issues in the data transfer.

#### 3.2.3 Baseline methods

We compared the proposed MUVINN-reg algorithm with two conventional image registration approaches: a parametric approach using Elastix^28^ and non-parametric approach with the diffeomorphic demons algorithm.^29^ For the comparison, we used the dataset of experiment 1 consisting of seven pairs of repeated scans: S1 and S2, S1 and S3, S1 and S4, S1 and S5, S1 and S6, and S1 and S7. For a fair comparison, images were preprocessed using Frangi vesselness filtering with same standard deviation values used for MUVINN-reg (σ={12,9,5,3,2}) and adaptive intensity modulation before image registration. Also, since the performance of these algorithms can highly depend on parameters, experimental tuning was performed to find a parameter configuration yielding accurate registration results in similar computational times of MUVINN-reg. For more information regarding the implementation and tuning of these methods, the reader can refer to section 2 in the Supplementary Material.

#### 3.2.4 Evaluation metrics

Peak signal-to-noise ratio (PSNR), NCC, Dice similarity coefficient (DSC), and target registration error (TRE) were computed to quantify performance of the proposed framework. While PSNR and NCC are based on similarity between image intensities,^30^ DSC and TRE measure the geometric overlap between vascular structures. DSC was computed between binary masks of vessel segmentation (performed on both fixed and moving images as described in Sec. 3.1.3.) before and after alignment. Regarding the TRE analysis, matching landmarks in correspondence of vessel branching points were manually annotated on both fixed and moving images using the open source software MeVisLab.^31^ After the co-registration, coordinates of the annotated points on the fixed image were transformed by feedforwarding them through the trained network into the moving coordinate system. Distances between corresponding points were computed before and after co-registration. Registration results were also evaluated qualitatively by plotting maximum intensity projections (MIPs) of the image pair overlay. The fixed image was encoded in the blue channel, whereas the moving one in the red channel. Overlapping structures appear as magenta. For better visualization of vascular features, images were processed using Frangi vesselness filtering and intensity adaptive modulation, similarly to previous studies.^4^^,^^7^^,^^24^^,^^25^

## 4 Results

### 4.1 Experiment 1: Performance of MUVINN-reg in Correcting Misalignments due to Breast Repositioning

**Fig. 2:**
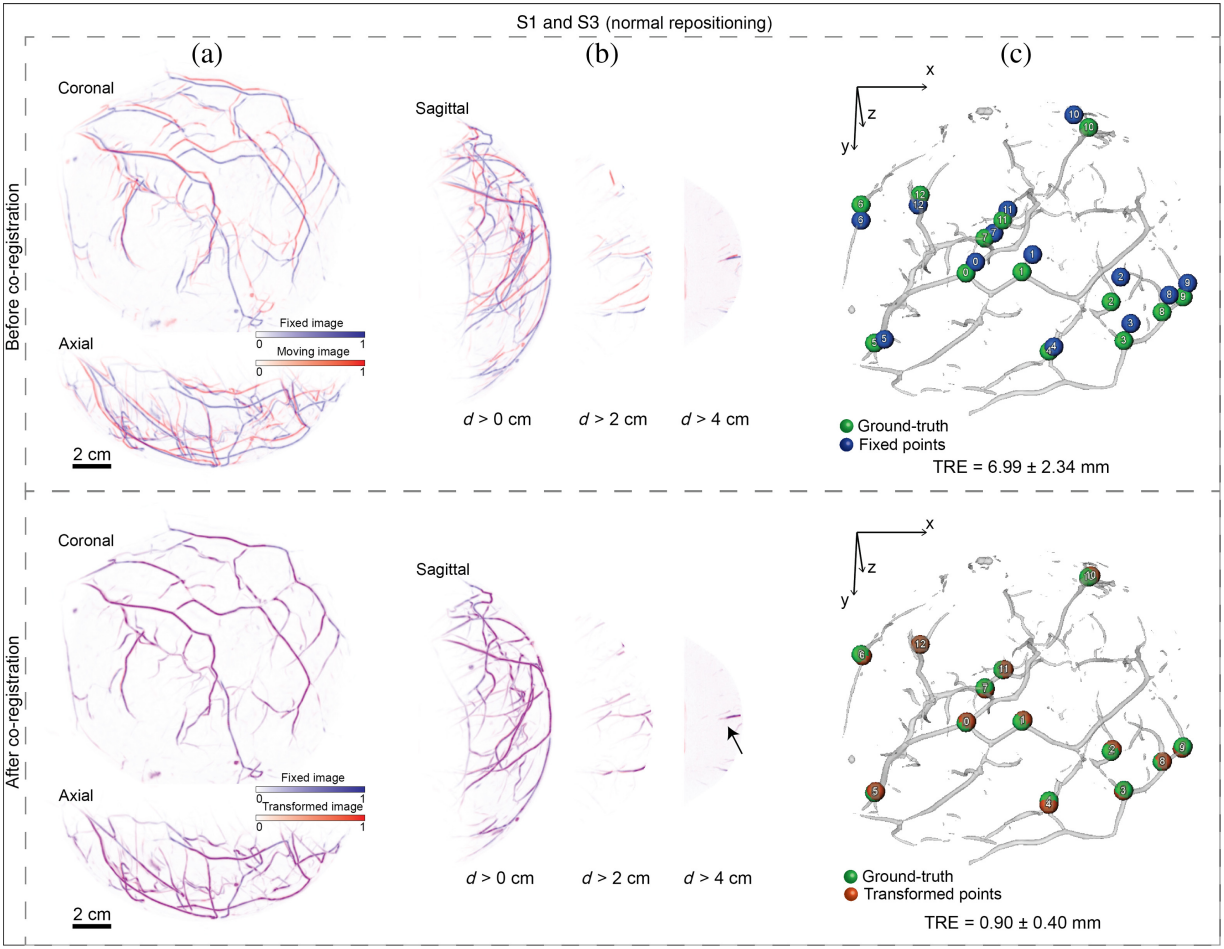
Performances of MUVINN-reg in correcting misalignments due to normal breast repositioning. (a) Coronal and axial MIPs of the overlay of pairs of PAT repeated scans (b) Sagittal MIPs of the overlay at different depths from the breast surface (>0, 2, and 4 cm). (c) 3D rendered vascular network of the moving image; before co-registration: green spheres represent the ground-truth, namely the annotated points on the moving image, and blue spheres are the annotated points on the fixed image; after co-registration: red spheres are the annotated points on fixed image after transformation to the moving coordinate system. The black arrow indicates the alignment of a vessel deeper than 4 cm. TRE is reported as mean and standard deviation of the distances between corresponding points.

Despite the use of breast supporting cups, misalignment of vascular structures always occurred after breast repositioning inside the recording aperture. In Fig. 2(a), MIPs of the overlay of pair S1 and S3 are shown. Misalignment was observed also in deeper regions of the breast, as shown in the depth-layered MIPs in Fig. 2(b). TRE analysis showed a displacement equal to 6.99±2.34  mm [Fig. 2(c)]. As shown in the bottom row of Fig. 2(a), co-registration by MUVINN-reg improved alignment of vascular features. Also, Fig. 2(b) shows the improvement in the alignment of a vessel deeper than 4 cm (pointed by the black arrows). After co-registration, TRE decreased to 0.90±0.40  mm, which represents a mean error of less than 2 voxels [bottom row of Fig. 2(c)]. Similar results were obtained also in other cases (Table 2).

**Table 2:** Quantitative results for each experiment.

	Before co-registration				After co-registration			
Pair	PSNR (dB)	NCC	DSC	TRE (mm)	PSNR (dB)	NCC	DSC	TRE (mm)
S1 and S2 (both 720 nm)	39.95	0.36	0.33	10.97 ± 3.78	44.01	0.73	0.63	0.89 ± 0.48
S1 and S3 (both 720 nm)	38.75	0.14	0.12	6.99 ± 2.34	44.40	0.75	0.69	0.90 ± 0.40
S1 and S4 (both 720 nm)	38.79	0.14	0.13	6.22 ± 1.79	44.80	0.78	0.69	0.64 ± 0.33
S1 and S5 (both 720 nm)	38.55	0.13	0.10	5.99 ± 1.23	44.85	0.78	0.69	0.63 ± 0.26
S1 and S6 (both 720 nm)	37.96	0.12	0.09	24.98 ± 6.75	41.84	0.58	0.48	1.97 ± 2.62
S1 and S7 (both 720 nm)	38.27	0.12	0.08	33.31 ± 5.16	41.43	0.51	0.43	2.33 ± 2.25
S1 (720 nm) and S2 (870 nm)	39.95	0.36	0.39	10.97 ± 3.78	43.57	0.69	0.64	0.75 ± 0.29
S1 (720 nm) and S3 (870 nm)	38.75	0.14	0.12	6.99 ± 2.34	43.80	0.71	0.66	0.92 ± 0.47
S8 (cup 7) and S11 (cup 5)	37.69	0.07	0.05	17.3 ± 3.71	42.82	0.68	0.49	0.82 ± 0.56
S8 (cup 7) and S12 (cup 4)	37.21	0.05	0.04	20.77 ± 2.78	42.45	0.67	0.43	1.32 ± 1.02
S8 (cup 7) and S13 (cup 4)	37.40	0.05	0.04	28.11 ± 5.25	41.79	0.60	0.41	1.45 ± 0.9
S8 (cup 7) and S14 (cup 8)	38.15	0.10	0.04	9.19 ± 2.26	43.42	0.71	0.59	1.30 ± 0.44
S8 (cup 7) and S15 (cup 8)	37.96	0.09	0.05	15.17 ± 3.23	42.27	0.62	0.54	0.81 ± 0.42

**Fig. 3:**
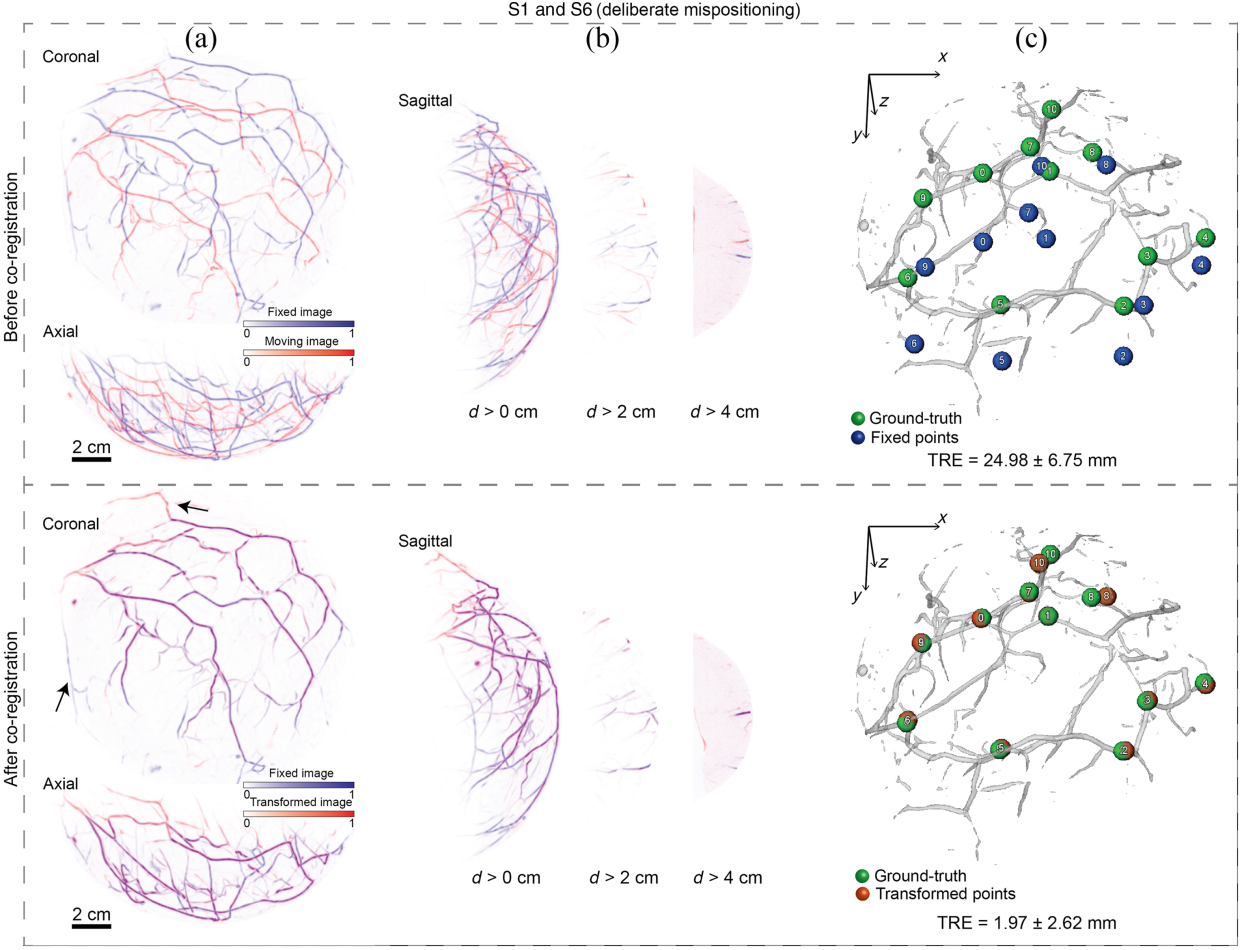
Performances of MUVINN-reg in correcting misalignments due to deliberate breast mispositioning. Refer to Fig. 2 for explanation. The black arrows show vessels missing in one of the two repeated scans due to the different positioning of the breast. TRE is reported as mean and standard deviation of the distances between corresponding points.

For deliberate mispositioning, the breast was intentionally mispositioned inside the cup to cause larger and more complex deformation. In fact, this produced a greater initial displacement (maximum of mean displacements equal to 33.31 mm compared to 10.97 mm for the normal repositioning as shown in Table 2). This was also prominent in the MIPs of the overlay [pair S1 and S6 before co-registration shown in Fig. 3(a)]. For the specific case of pair S1 and S6, TRE analysis resulted in an initial displacement of 24.98±6.75  mm. Despite the larger displacement, MUVINN-reg was able to successfully co-register this pair of scans. MIPs of the overlay showed the presence of vessels missing in one of the two scans due to different positioning of the breast inside the cup [shown by the black arrows in Fig. 3(a), after co-registration]. TRE analysis showed a decrease of the displacement to 1.97±2.62  mm. In general, for every case of experiment 1, we observed an improvement of all the metrics from before to after co-registration (Table 2).

### 4.2 Experiment 2: Testing MUVINN-reg with Different Illumination Wavelengths

**Fig. 4:**
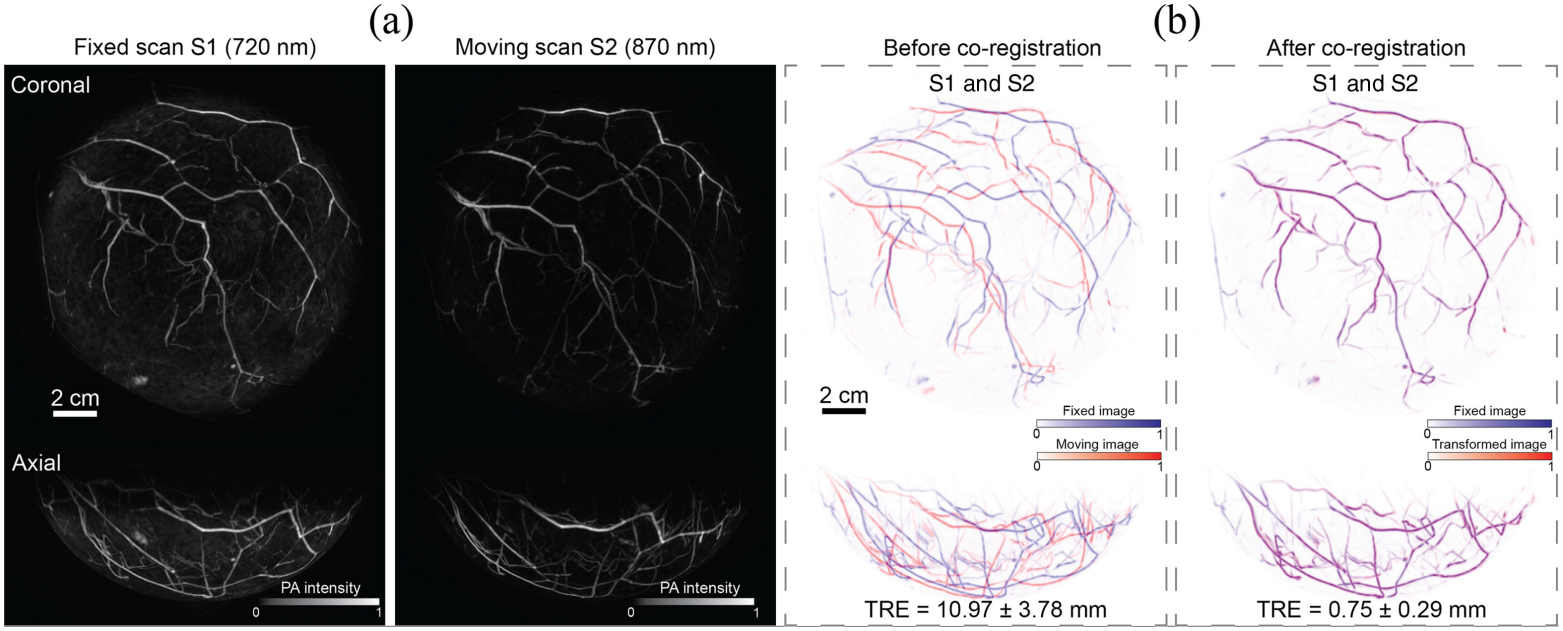
Performances of MUVINN-reg in co-registering repeated PAT scans with different illumination wavelengths. (a) MIPs of fixed and moving PAT images for pair S1 and S2. (b) Coronal and axial MIPs of the overlay of pairs of PAT repeated scans showing misalignment before and after co-registration. TRE is reported as mean and standard deviation of the distances between corresponding points.

For this experiment, scan S1 at 720 nm was defined as fixed, and scans S2 and S3 at 870 nm as moving. Figure 4 shows the performance of MUVINN-reg in co-registering pair S1 and S2. MIPs of PAT scans [shown in Fig. 4(a)] show differences in intensity due to different absorption of tissue chromophores at the two wavelengths 720 and 870 nm (e.g., skin intensity is higher in the 720 nm PAT scan due to higher absorption of melanin). To enable accurate comparison between co-registered images, the displacement field represented by the network was applied to the corresponding 720 nm version of the moving scans. MIPs of the overlays before and after co-registration are shown in Fig. 4(b). As a result, the proposed framework was able to successfully co-register PAT scans with different illumination wavelengths and decreasing the TRE from 10.97±3.78  mm and 6.99±2.34  mm to 0.75±0.29  mm and 0.92±0.47  mm, respectively for pairs S1 and S2 as well as S1 and S3. Values of displacement after co-registration were similar to those obtained in experiment 1 for pairs S1 and S2 (both 720 nm) as well as S1 and S3 (both 720 nm) showing the robustness of MUVINN-reg to different illumination wavelengths. Image similarity always increased from before to after co-registration (see Table 2).

### 4.3 Experiment 3: Testing MUVINN-reg with Different Breast Cup Sizes

The Twente PAM3 uses breast-supporting cups, which help to stabilize the breast during the image acquisition. The selection of the appropriate cup size is determined by the breast size, which is assessed through a fitting process prior to the acquisition. If the cup is inadvertently chosen smaller than the breast, the outer regions of the breast closer to the chest wall will extend beyond the cup and fall out of the field-of-view. On the other hand, choosing a cup bigger than the breast will not stabilize the breast appropriately. Although it is unlikely that different cups would be chosen for repeated measurements during NAC monitoring, it is still possible for the breast to undergo morphological changes that would alter the field-of-view. So, it is important to test the capabilities of the framework in case of field-of-view inconsistency between scans.

**Fig. 5:**
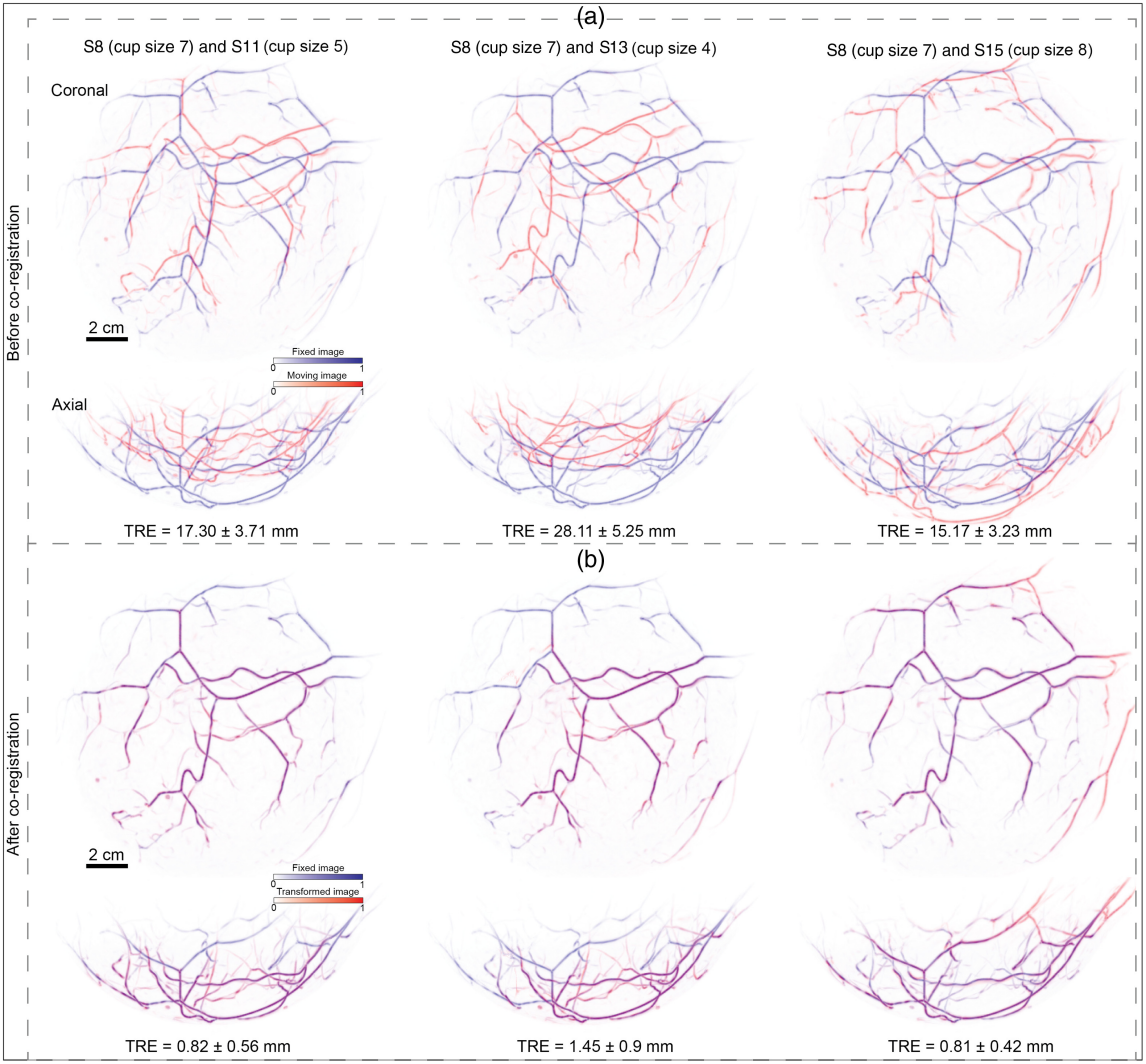
Performances of MUVINN-reg in co-registering repeated PAT scans with different breast-supporting cups. (a) MIPs of the overlay before co-registration for pairs S8 (cup size 7) and S11 (cup size 5), S8 (cup size 7) and S13 (cup size 4), as well as S8 (cup size 7) and S15 (cup size 8). (b) MIPs of the overlay after co-registration for the same pairs. TRE is reported as mean and standard deviation of the distances between corresponding points.

Figure 5 shows the MIPs of overlay of the fixed image S8 (cup size 7) with three scans acquired with different cup sizes: (a) S11 (cup size 5), (b) S13 (cup size 4), (c) S15 (cup size 8) before [panel (a)] and after [panel (b)] co-registration by MUVINN-reg. TRE analysis showed larger initial displacements than the other experiments. MUVINN-reg was able to retrieve the deformation field accurately for each pair. After co-registration, from the MIPs of overlay, the inconsistency of field-of-view between different cup sizes was clearer to notice: for pairs S8 and S11 as well as S8 and S13, deeper vascular structures of the breast were mostly blue due to their absence in the smaller cup scan [see Fig. 5(b)]. Comparing S8 and S15 after co-registration, vessels closer to the chest wall were imaged in S15 and not in S8. TRE analysis after co-registration showed a decrease of the displacement from 17.3±3.71  mm, 28.11±5.25  mm, and 15.17±3.23  mm to 0.82±0.56  mm, 1.45±0.9  mm, and 0.81±0.42  mm, respectively, for pairs S8 and S11, S8 and S13, as well as S8 and S15, respectively. Also for this experiment, the result of the quantitative analysis is an increase of all similarity metrics (Table 2).

### 4.4 Comparison with Baseline Methods

**Fig. 6:**
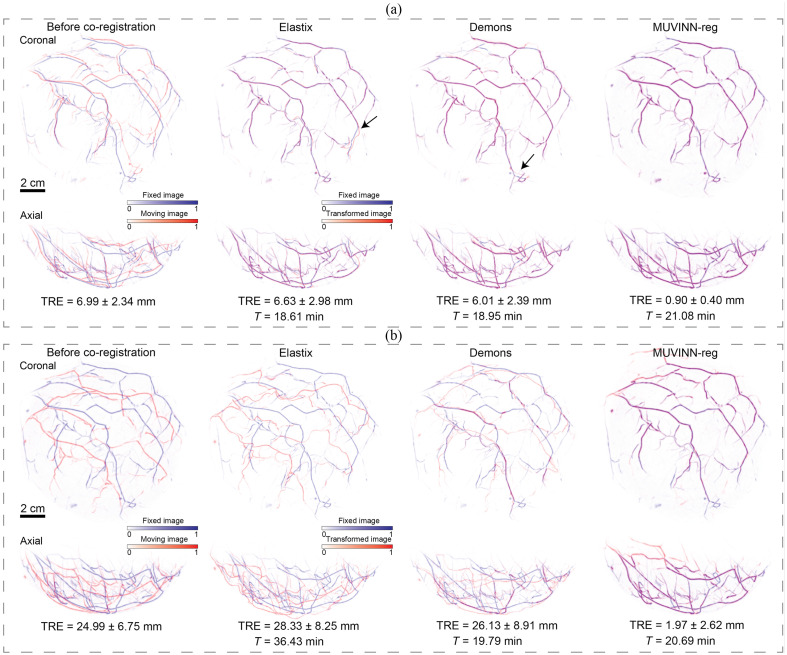
Comparison between parametric approach using Elastix, non-parametric diffeomorphic Demons algorithm, and the proposed MUVINN-reg. (a) Coronal and axial MIPs of the overlay before co-registration and after co-registration using Elastix, Demons and MUVINN-reg for pair S1 and S3 (normal breast repositioning). The black arrows show regions with misalignment of vascular structures. (b) Coronal and axial MIPs of the overlay before co-registration and after co-registration using Elastix, Demons, and MUVINN-reg for pair S1 and S6 (deliberate breast mispositioning). TRE and computational time (T) are reported.

Optimal configuration of parameters was selected for each baseline method after experimental tuning (data shown in section 2 in the Supplementary Material). Figure 6 shows the qualitative comparison of MIPs of the overlay after co-registration by Elastix, Demons, and MUVINN-reg for two pairs of repeated PAT scans: S1 and S2 (normal repositioning) as well as S1 and S6 (deliberate mispositioning). Although, for pair S1 and S2 (normal repositioning), major vascular structures were found to be aligned after co-registration, the black arrows point to regions where misalignment of vascular structures was present. In contrast, this was not the case for MUVINN-reg. Regarding the S1 and S6 pair (deliberate mispositioning), both Elastix and Demons were not able to correctly align the main vascular structure, in contrast to our proposed algorithm. The weak performance of the parametric approach by Elastix might be attributed to two main factors: (1) the parameterization of the transformation model (rigid + B-spline) may not be appropriate for the deformation that occurs in the case of breast repositioning, and (2) a too low number of iterations in order to achieve the global minimum. Regarding to the nonparametric approach by Demons, one possible reason could be the use of an intensity-based metric such as the sum of squared differences that does not take into account variations in intensity between the two images due to different light distribution. Regarding computational times, these are heavily dependent on the parameter selection (mainly the number of iterations and the stopping condition if present). To avoid excessively long computational times, only configurations with a reasonable number of iterations were tested (see section 2 in the Supplementary Material). However, with the selected configurations of parameters, MUVINN-reg showed better performance at the cost of slightly higher computational times (e.g., pair S1 and S3, MUVINN-reg: 21.08 min, Elastix: T=18.61  min, Demons: T=18.65  min).

## 5 Discussion

Promising developments have been presented in the research into PA imaging for NAC monitoring.^6^^,^^7^ However, the analysis of longitudinal PA images in this setting has hitherto been qualitative. Issues related to organ repositioning in the imaging volume cannot be discounted, which can result in nonoptimal correlation between images and consequently complications in their comparison to extract subtle biological changes. Specifically, repositioning affects the position of vascular structures relative to the coordinate system of the device, posing challenges to identify the same structure-of-interest across multiple serial images. The use of a breast-supporting cup makes the repositioning procedure more reproducible^9^ as it provides a rigid containment structure for the breast. Nevertheless, the present study showed that despite the use of cups, mean displacements in the range of 5.99 to 10.97 mm were found between images.

To address these problems, we developed MUVINN-reg, a framework that uses coordinate-based neural networks and multiscale Frangi vesselness filtering to co-register longitudinal PAT scans in three dimensions. The effectiveness of the framework was investigated in correcting for geometric misalignments that could potentially take place in subject repositioning. MUVINN-reg demonstrated a drastic reduction of displacement and increase in image similarity after co-registration for several tested scenarios (Table 2).

The use of a coordinate-based neural network makes the proposed registration framework unique in that it is capable of being able to represent deformation fields in a continuous domain. This allows the deformation field to be known at any coordinate in the domain and avoids the use of finite-differences techniques to calculate spatial derivatives of the deformation field.^17^ In addition, the approach gives the possibility to integrate similarity metrics, tailored for the image registration problem, as long as they are differentiable. For the specific case of PA images, using a similarity term based on vesselness features allows only relevant structures within images to be considered, and potential noise to be excluded. This feature combined with the use of NCC allows the method to be robust to variations in image intensities (as shown in Fig. 4 for experiment 2), which might occur in repeated PA imaging due to changes in light distribution. The implementation of a coarse-to-fine strategy is critical to the success of the framework. As already shown in other studies,^15^ decomposing the image in different scales facilitates the registration process, which is the reason why image registration frameworks often work on multiple pyramidal levels. We observed that the use of a coarse-to-fine strategy crucially improves accuracy and consistency of the framework (see section 1 in the Supplementary Material). Finally, the contribution of the Jacobian regularization in the loss function helps to represent regular deformation fields and avoids that vessels are excessively shrunk or expanded. The combination of all these features makes the algorithm capable of co-registering pairs of images at different displacement magnitudes (experiment 1), affected by inconsistencies in image intensity (experiment 2), and field-of-view (experiment 3).

In experiment 3, the algorithm was subjected to pairs of images with different fields-of-view, where some vessels were present in only one of the two images. This is important in the context of NAC monitoring as it is very hard to image the same breast volume in longitudinal imaging. There is also potential utility of the algorithm in image mosaicking or image stitching,^28^ in scanning imaging systems where multiple smaller and overlapping images are compounded. Here, MUVINN-reg can be applied to perform the mosaicking based on overlapping regions.

Comparison with baseline methods showed the superiority of MUVINN-reg over the existing conventional image registration algorithms based on parameterized transformations and intensity-based metrics. Adaption of these methods to PA imaging would require more intensive image preprocessing and accurate parameter tuning to cope with the sparse nature of PA data, and the complexity of the deformation fields.

One limitation of the proposed framework lies in the necessity of parameter tuning to ensure optimal performance. The most critical parameters are the number of iterations and the values of the Frangi scales during the optimization. A higher number of the iterations gives better results in terms of co-registration accuracy but leads to higher computational times. Regarding the choice of the scales, higher standard deviations produce more regular displacement fields with the risk of not aligning fine structures in the image. On the other hand, the use of very low standard deviations lead to the risks of noise being considered in the optimization process which would ultimately produce wrong displacement fields and irregular distortion of structures in the transformed image. Also, starting with larger σ values allows to mitigate the sparsity of the image, therefore improving gradients for the network optimization. At this stage, parameter selection was performed using a trial and error approach and a good parameter configuration was found which can work in every scenario. However, the presence of inaccuracies in the alignment of some vessels was noted in certain cases (Fig. S3 in the Supplementary Material). We believe this is because the misalignment of these small vascular structures did not have a significant contribution to the loss function during optimization. This can be solved by increasing either the number of iterations or the number of points sampled in each iteration, at the cost of increasing the computational expenses. Also, in future, automated parameter optimization and a user-friendly interface will be implemented to make the framework usable in a practical setting. Another limitation lies in computational time. While the fact that INRs are self-supervised techniques is a big advantage because they do not require a training set, each pair of images requires optimization of a new neural network. This results in higher computational times compared to a supervised technique. The computational time for each pair of image was 20.42±0.56  min. These are acceptable computational times when considering the context of disease or treatment monitoring but become less convenient in the case of real time applications.

Finally, the effects of NAC on the patient’s breast can be heterogeneous, and it is largely unknown how the therapy changes the morphology of the breast and blood vessels. For this reason, it is essential to evaluate the framework in actual treatment monitoring conditions. It is worth noting that the dataset at this point is limited to only one PAT device with its own recording aperture geometry and breast-supporting system. We encourage researchers in the community to consider applying our method to their own image datasets for further validation and exploration.

## 6 Conclusions

We presented MUVINN-reg, an automatic 3D image registration framework that can address the challenges of geometric misalignment in longitudinal PAT breast scans. We demonstrated the robust performance in co-registering images under different unfavorable repositioning scenarios. MUVINN-reg can align vessels deeper than 4 cm. This advancement holds significant promise for enabling reproducible and quantitative monitoring of disease progression and treatment response in breast cancer using PAs.

## Data Availability

All data and codes presented in this study are publicly available in the following GitHub repository: https://github.com/brunodesanti/muvinn-reg
