# Supplementary material for: Automated three-dimensional image registration for longitudinal photoacoustic imaging
Source: J Biomed Opt. 2024 Jan 13;29(Suppl 1):S11515. doi: 10.1117/1.JBO.29.S1.S11515 (PMC10787589; doi:10.1117/1.JBO.29.S1.S11515)
Supplement: Supplementary file 1 [file JBO_029_S11515_SD001.pdf]

Supplementary Material for:
Automated three-dimensional image registration for longitudinal photoacoustic imaging

Bruno De Santi[1,*], Lucia Kim[1], Rianne F. G. Bulthuis[1,2], Felix Lucka[3], Srirang Manohar[1]

[1]Multi-Modality Medical Imaging group, TechMed Centre, University of Twente, Enschede, 7500 AE, The Netherlands
[2]Department of Radiology, Medisch Spectrum Hospital, Enschede, 7512 KZ, The Netherlands.
[3]Centrum Wiskunde en Informatica (CWI), Amsterdam, 1098 XG, The Netherlands
[*]*b.desanti@utwente.nl*

1. Effect of coarse-to-fine optimization

As described in Sec. 3.1.1, the original implementation of the use of implicit neural representations for image registration (Ref. 17 in the manuscript) was adapted to work on photoacoustic images mainly by introducing a coarse-to-fine strategy. For comparison, the original algorithm was applied to all pairs of experiment 1 with normal repositioning using same network hyperparameters. The only differences were that: 1) Fixed and moving images were preprocessed only once before registration using multiscale Frangi vesselness filtering with the following standard deviation values ($\sigma = \{7, 5, 3, 1.5\}$[1]) and 2) a fixed side length of the local patch around sampled points equal to 0.05 was used. Fig. S1 shows the results on the representative pair *S1-S5*, where the algorithm failed to retrieve the correct deformation field. Maximum intensity projections of the overlays [Fig. S1(a)] after co-registration showed wrong alignment of vascular structures, despite the decrease observed in the training loss curve [Fig. S1(b)]. The represented displacement field resulted to be highly irregular, as shown in Fig.S1(c), which is a coronal projection of the magnitude of the displacement field. Similar results were observed for pair *S1-S2* (data not shown), while the algorithm was able to retrieve a correct deformation field for the other three remaining pairs. The main reason for this low consistency is due to the presence of

[1] We found that the configuration $\sigma = \{12, 9, 5, 3, 2\}$ allows to accurately register every pair of images under all the different scenarios (normal repositioning, mispositioning, different illumination wavelengths and different breast-supporting cup). This is the reason why we chose that configuration as default, and presented this in the paper. Nonetheless, for displacements with a small low-frequency component, such as normal breast repositioning using a cup, using $\sigma = \{7, 5, 3, 1.5\}$ is enough to correctly represent the deformation field, reason why, we chose this configuration for this additional experiment.

features at different scales within the image, so that the network attempts to optimize the deformation field in multiple small regions of the image with different levels and scales of deformation. For this reason, we considered to implement a coarse-to-fine approach that would allow the network to gradually optimize structures at different scales. Fig. S2 shows the effects of using such an approach on the *S1*-*S5* pair. Starting from the top, each row shows the optimization results for each sigma. Fig. S2(a) shows the local patches on which the normalized cross correlation is calculated, whose size depends on the current sigma. Fig. S2(b) shows the training loss curve, where it is interesting to note how just the few first iterations at sigma equal to 7 are enough to drastically reduce the loss. By the end of the first 5000 iterations, there is already an improvement in image alignment [Fig. S2(c) and (d)] and a coarse and regular representation of the displacement field [Fig. S2(e)]. Subsequently, by progressively decreasing the sigma value, smaller sized features will be enhanced in the image that will contribute to the loss function and thus enable local refinement of the displacement field representation.

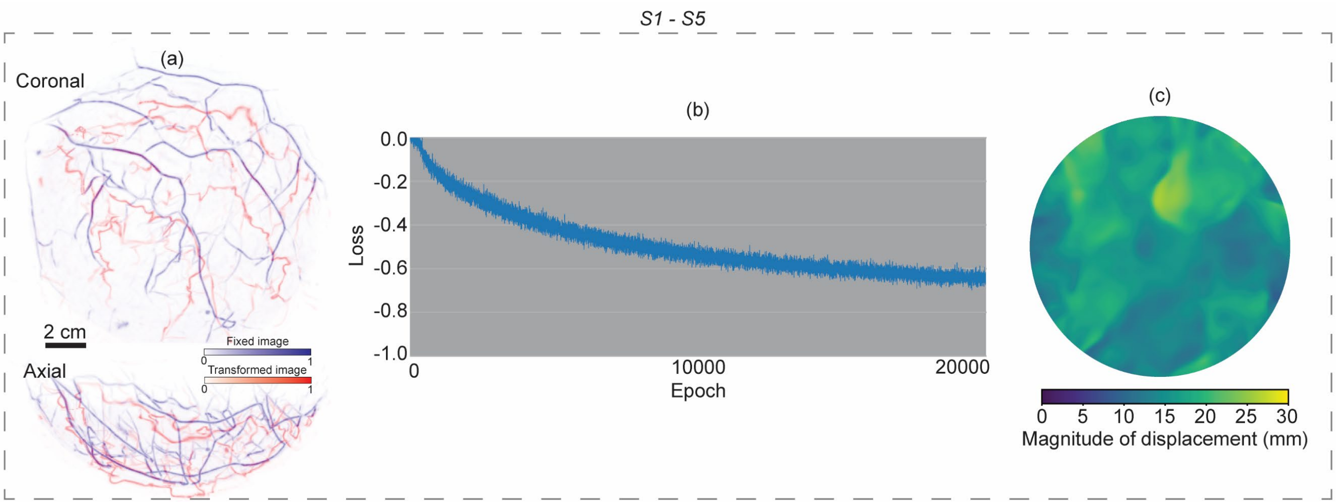

Fig. S1 Registration results on pair *S1*-*S5* without coarse-to-fine strategy. (a) Maximum intensity projections of the overlay after co-registration; (b) Training loss curve; (c) Coronal projection of the magnitude of the displacement field.

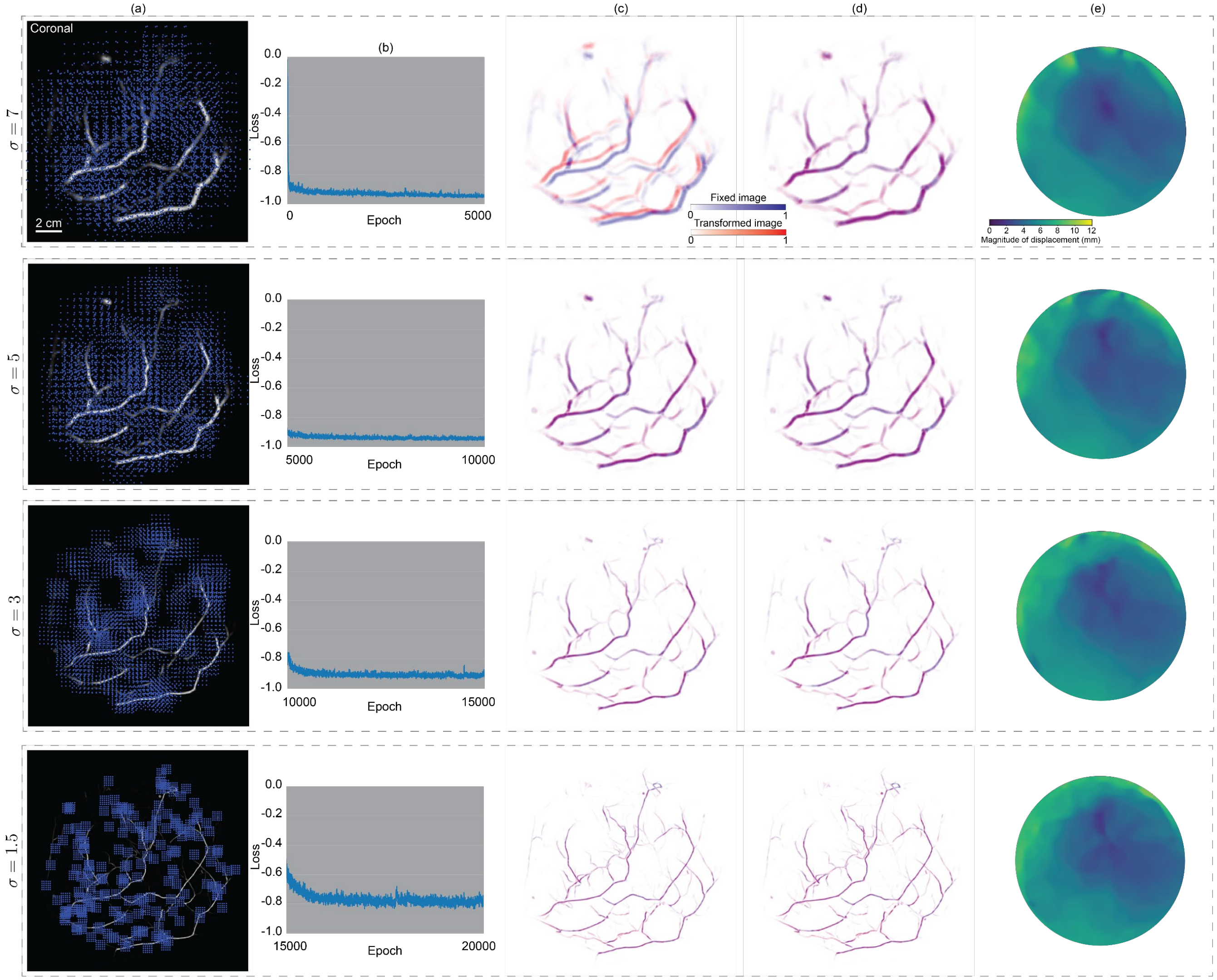

Fig. S2 Visualization of the coarse-to-fine strategy on co-registering pair *S1-S5*. (a) Point sampling in the fixed image domain; (b) Training loss curve; (c) Maximum intensity projections of the overlay at the beginning of the current phase; (d) Maximum intensity projections of the overlay at the end of the current phase; (e) Coronal projection of the magnitude of the displacement field.

2. Comparison with other image registration approaches

We compared the proposed algorithm with two conventional image registration approaches: a parametric approach using Elastix (ref. 28 in the manuscript), and non-parametric approach with the diffeomorphic demons algorithm (ref. 29 in the manuscript). For the comparison, we used the dataset of experiment 1 consisting of seven pairs of repeated scans: *S1-S2, S1-S3, S1-S4, S1-S5, S1-S6* and *S1-S7.* For a fair comparison, images were preprocessed using Frangi vesselness filtering with same standard deviation values used for MUVINN-reg ($\sigma = \{12, 9, 5, 3, 2\}$) and adaptive intensity modulation

before image registration. Also, since the performance of these algorithms can highly depend on parameters, experimental tuning was performed to find a parameter configuration yielding accurate registration results in similar computational times of MUVINN-reg.

2.1 Parametric approach using Elastix

Elastix follows a classical parametric registration scheme, therefore, an optimization procedure is performed in order to find the optimal parameters of a transformation model. This approach was implemented by using the Simple Insight Toolkit class *itk::simple::ElastixImageFilter*. A rigid transformation followed by a non-rigid B-spline were chosen as transformation models to correct for both linear and non-linear deformations. Sampling was performed inside the cups, to optimize transformation only inside the breast. Six different configurations of parameters were tested by changing number of iterations for the rigid registration (N_{rig}), number of iterations for the non-rigid registration ($N_{non-rig}$) and the rigidity penalty (α_{rig}). These configurations are listed in Table S1.

Table S1 Configurations of parameters for Elastix.

Configuration	N_{rig}	$N_{non-rig}$	α_{rig}
E0	400	1200	0.5
E1	400	1200	1
E2	800	1600	0.5
E3	800	1600	1
E4	400	800	0.5
E5	400	800	1

A multiresolution scheme with 4 pyramid levels was used with number of iterations for each pyramid equal to N_{rig} for rigid and equal to $N_{non-rig}$ for B-spline. Finally, similarity term of loss function was the *AdvancedNormalizedCorrelation* and the *TransformRigidityPenalty* with weight equal to α_{rig} was used as regularization.

2.2 Non-parametric diffeomorphic Demons

Contrary to parametric approaches, this method does not require parametrization of the transformation model but instead optimizes a global energy based on voxel correspondences (ref. 29 in the manuscript). An efficient and open source implementation available in the Simple Insight Toolkit was used, *itk::simple::FastSymmetricForcesDemonsRegistrationFilter*. The algorithm was applied on a multipyramidal scheme: optimization starts with a downsampled (rescaling factor equal to 6) and smoothed (standard deviation equal to 12) version of the images and then images are progressively upsampled to their original scale in 4 steps. Six different configurations of parameters were tested by changing number of iterations (N) and the smoothing standard deviation (σ). These configurations are listed in Table S2.

Table S2 Configurations of parameters for Demons.

Configuration	N	σ
D0	250	0.5
D1	500	0.5
D2	1000	0.5
D3	250	1
D4	500	1
D5	1000	1

2.3 Results and discussion

Table S3 shows mean and standard deviation values of TRE for each tested configuration using Elastix and Demons. Configuration E5 led to the lowest average TRE (TRE = 6.96 ± 1.87 mm) for Elastix. However, using configuration E5 the ITK error “Too many samples map outside moving image buffer” occurred during the registration process of pair *S1 - S6* and *S1 - S7*. Same error occurred for E4 when registering pair *S1 – S7*. We decided to choose configuration E2 as final, which reported the lowest TRE values (13.92 ± 9.79 mm) and could actually register every image pair.

For Demons, configuration D1 was the best for Demons with TRE = 14.53 ± 11.39 mm.

Table S3 Mean and standard deviation values of target registration error (TRE) for each configuration of parameters using Elastix and Demons. T = computational time. *For E4, the following ITK error: "Too many samples map outside moving image buffer" occurred when registering pair *S1 – S7*. **For E5 the same error occurred for pairs *S1 - S6* and *S1 - S7*.

Method	Configuration	TRE (mm)	T (minutes)
**Elastix**	**E0**	15.09 ± 11.79	16.22 ± 4.51
	**E1**	15.21 ± 11.43	14.78 ± 4.25
	**E2**	13.92 ± 9.79	23.30 ± 8.11
	**E3**	14.06 ± 10.02	23.52 ± 8.39
	**E4***	11.31 ± 8.85	10.04 ± 2.24
	**E5****	6.96 ± 1.87	9.37 ± 0.34
**Demons**	**D0**	14.59 ± 11.65	9.54 ± 0.46
	**D1**	14.53 ± 11.39	19.13 ± 0.74
	**D2**	14.77 ± 11.60	38.96 ± 1.22
	**D3**	14.57 ± 11.51	11.89 ± 0.39
	**D4**	14.74 ± 11.73	23.03 ± 0.49
	**D5**	15.32 ± 12.44	46.02 ± 0.72

Table S4 shows the quantitative comparison among methods. MUVINN-reg resulted to be superior in co-registering PAT repeated scans. But, interestingly, Demons reported higher values of image similarity metrics than MUVINN-reg. However, higher metrics do not necessarily mean better registration, since these metrics are more sensitive to noise and alignment of smaller vessels have a low contribution. When comparing TRE values and overlap between vascular structures (Dice similarity coefficient), MUVINN-reg showed better registration performances.

Table S4 Quantitative comparison with parametric approach using Elastix and non-parametric diffeomorphic Demons algorithm. Refer to Table 2 for metrics before co-registration. Acronyms: PSNR = peak signal-to-noise ratio, NCC = normalized cross correlation, DSC = Dice similarity coefficient, TRE = target registration error, T = computational time.

	**Parametric approach (Rigid + Bspline) - Elastix**					**Non-parametric diffeomorphic Demons algorithm**					**MUVINN-reg (proposed)**				
**Pair**	**PSNR (dB)**	**NCC**	**DSC**	**TRE (mm)**	**T (minutes)**	**PSNR (dB)**	**NCC**	**DSC**	**TRE (mm)**	**T (minutes)**	**PSNR (dB)**	**NCC**	**DSC**	**TRE (mm)**	**T (minutes)**
S1 – S2	39.58	0.28	0.45	9.07 ± 3.90	17.02	46.63	0.86	0.61	9.06 ± 4.07	17.81	44.01	0.73	0.63	0.89 ± 0.48	20.86
S1 – S3	41.21	0.49	0.64	6.63 ± 2.98	18.61	47.38	0.89	0.66	6.01 ± 2.39	18.95	44.40	0.75	0.69	0.90 ± 0.40	21.08

S1 – S4	40.11	0.33	0.47	4.39 ± 1.42	17.18	47.85	0.90	0.67	4.3 ± 1.55	19.79	44.80	0.78	0.69	0.64 ± 0.33	20.98
S1 – S5	40.04	0.34	0.55	8.23 ± 3.24	17.68	48.66	0.92	0.74	7.48 ± 2.93	18.66	44.85	0.78	0.69	0.63 ± 0.26	20.83
S1 – S6	38.26	0.13	0.07	28.33 ± 8.25	36.43	43.30	0.66	0.27	26.13 ± 8.91	19.79	41.84	0.58	0.48	1.97 ± 2.62	20.69
S1 – S7	38.60	0.11	0.07	26.86 ± 2.95	32.88	42.64	0.59	0.14	34.2 ± 4.39	19.78	41.43	0.51	0.43	2.33 ± 2.25	21.14

3. Examples of MUVINN-reg inaccuracies

Fig. S3 shows the examples of inaccuracies of MUVINN-reg mentioned in the Discussion section.

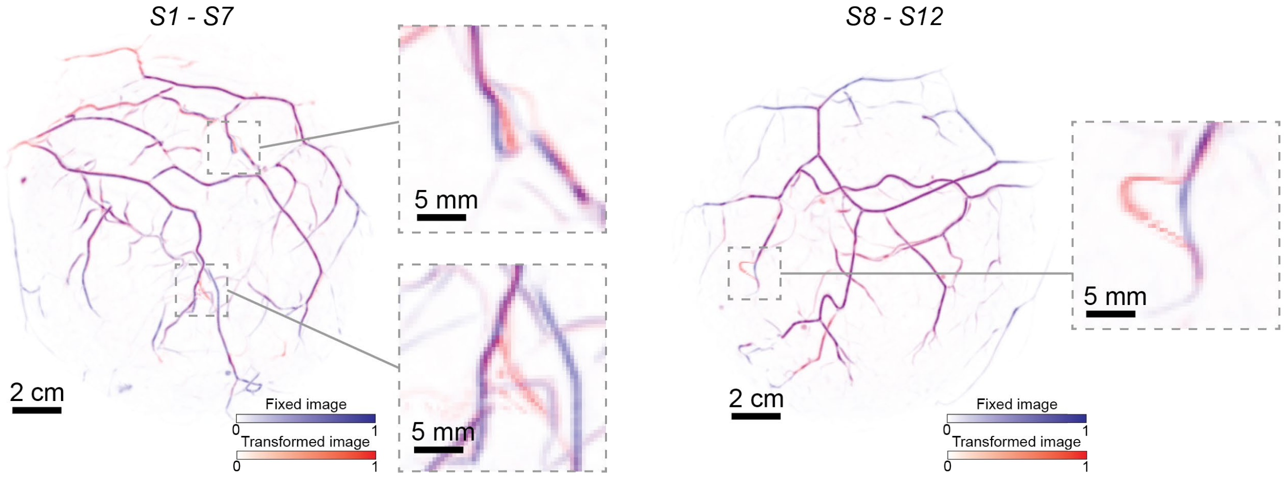

Fig. S3 Examples of MUVINN-reg inaccuracies. Regions indicated by dashed rectangles show regions with inaccurate vascular alignment.

4. Vascular segmentation with adaptive thresholding

The adaptive threshold map (T) was computed as following:

$$T(x) = T_f + (T_i - T_f) \cdot e^{-\tau D(x)}$$

where $D(x)$ is the depth map which represents the Euclidean distance in cm of each voxel from the cup surface. The parameters T_i, T_f and τ are fixed parameters which represent the threshold at the cup surface, the threshold at the deepest point inside the cup, and the exponential decay rate, respectively. For our images, $T_i = 0.008, T_f = 0.003$ and $\tau = 100$ showed satisfactory results.
